# Animal Welfare and Food Safety When Slaughtering Cattle Using the Gunshot Method

**DOI:** 10.3390/ani12040492

**Published:** 2022-02-16

**Authors:** Jan Hultgren, Katrin J. Schiffer, Jakub Babol, Charlotte Berg

**Affiliations:** 1 Department of Animal Environment and Health, Swedish University of Agricultural Sciences, P.O. Box 234, 53223 Skara, Sweden; katrin.schiffer@lansstyrelsen.se (K.J.S.); lotta.berg@slu.se (C.B.); 2 Department of Biomedical Science and Veterinary Public Health, Swedish University of Agricultural Sciences, P.O. Box 7036, 75007 Uppsala, Sweden; jakub.babol@slu.se

**Keywords:** brain haemorrhage, on-farm slaughter, rifle, steer, slaughter hygiene, stun quality

## Abstract

**Simple Summary:**

Transporting cattle from the farm to the slaughterhouse is often stressful for the animal. With the gunshot method, it is stunned using a rifle while together with familiar herd members in an enclosure on the farm. The shot makes the animal unconscious. Then, as in normal slaughter, the animal is bled to death. Finally, it is transported to a nearby slaughterhouse. We aimed to assess the consequences for animal welfare and food safety of using the gunshot method. Twenty Hereford steers were shot with a hunting rifle using small-calibre ammunition from an elevated position and distance of 6–12 m. Each time, only one out of four to seven animals in a 16 × 10 m corral was shot. Based on the animals’ behaviour and physiological blood values, stress levels before shooting were low. Eleven animals were considered completely unconscious, while seven showed some signs of consciousness and two were poorly stunned. Two animals were reshot with heavier ammunition. Bleeding was satisfactory and little or no contamination was found on the carcasses. We conclude that the gunshot method is applicable to large beef steers while maintaining a satisfactory level of animal welfare and food safety, provided that the necessary conditions can be achieved.

**Abstract:**

Transporting cattle from farm to slaughterhouse is often stressful for the animal, which can impair the meat quality. With the gunshot method, the animal is stunned with a rifle shot while together with familiar herd members in their home environment, exsanguinated and transported to a nearby slaughterhouse. Aiming to assess the consequences for animal welfare and food safety, 20 Hereford steers aged 18–54 months were shot with .22 Magnum ammunition from an elevated position and distance of 6–12 m. Each time, only one out of four to seven animals in a 16 × 10 m corral was shot. Dressing was done on farm. Based on the animals’ behaviour and blood concentrations of cortisol, glucose and lactate, stress levels before shooting were low. Eleven animals were deeply stunned, the consciousness of seven others was ambiguous, and two were poorly stunned. Two animals were reshot. The bleed-out was satisfactory for all animals, and little or no faecal contamination was found on the carcasses. We conclude that the gunshot method is applicable to large beef steers while maintaining a satisfactory level of animal welfare and food safety, provided that the necessary conditions can be attained.

## 1. Introduction

Cattle are usually transported by road to a slaughterhouse where they sometimes spend the night before being slaughtered. Animals that have been raised in extensive outdoor conditions and rarely handled by humans can react strongly and become stressed when they are driven, crowded, transported and restrained [1,2,3,4,5,6]. Rough handling, exhaustion and injuries to slaughter animals can also lead to a poor meat texture and flavour, high ultimate pH and reduced slaughter yield, as well as a short shelf life of products [7,8,9]. In addition, animal handling is associated with the spread of infectious diseases [5,10] and injuries to handlers [11,12].

European Union (EU) Council Regulation (EG) 1/2005 on animal transport stipulates that no animal shall be transported unless it is ‘fit for the intended journey’. Sick and injured animals must not enter the human food chain. The Federation of Veterinarians of Europe [13] considered that the transport of live animals to slaughter should, where possible, be replaced by the transport of carcasses, and the World Organisation for Animal Health recommends that animal transport times be kept to a minimum [14].

Some of the problems associated with animal transport in connection with slaughter may be avoided by on-farm slaughter [15]. This may even be a reasonable alternative for small farms [16], when animals are slaughtered in small numbers or when they are unfit for transport. In many industrialized countries, farmers may also seek alternatives to centralized large-scale slaughter because they aim at a high level of control over the entire production chain as a way to improve animal welfare and product quality, and thereby achieve market benefits [17].

Cattle are most commonly stunned by a captive bolt that is propelled by a gunpowder cartridge or compressed air, but a rifle with a free bullet is sometimes used. The weapon is aimed at the forehead, even if rifles are fired at some distance, while the animal is situated in the stun box. All mechanical stunning requires that the animal is sufficiently immobilized for the shot to be positioned correctly. A successful shot results in almost immediate unconsciousness.

Unsatisfactory stunning is not uncommon at large-scale slaughter. Gregory et al. [18] reported inadequate stuns in 14–16% of the bulls shot with a captive bolt gun, compared to 5–7% of the steers and 5–6% of the heifers. At German, Swiss and Austrian slaughterhouses, 9% of cattle shot with a captive bolt gun were incompletely stunned [19]. At a Swedish slaughterhouse, inadequate stuns were most often seen in bulls and calves [20], with 17% of the 585 bulls studied showing signs of some degree of consciousness. Slaughterhouses with effective pneumatic bolt guns and equipment for restraint have also reported practical problems, since, even if the safety of the shot and the stunning effect are good, the restraint can result in severe stress [21]. Gregory et al. [18] noted that a faulty stun was more common in aroused cattle (19%) than in calm animals (8%). If the stun is found to be inadequate, the animal must be reshot immediately. This is usually the case in only a small percentage of animals [20].

During recent decades, there has been a great deal of interest in small-scale slaughter. To improve animal welfare before slaughter, the animal can be stunned at a distance with a rifle, while being kept with familiar herd members in a corral in their home environment, without previous stressful interactions with humans, followed by the exsanguination and transport of the carcass to a slaughterhouse. This has been called ‘the gunshot method’ [22,23]. Thus, the gunshot method may facilitate extensive grazing of beef cattle, which can promote the animals’ health [24] and nature conservation [25].

In Germany, researchers have investigated the practical considerations and consequences for animal welfare and meat quality of using the gunshot method in small beef breeds such as Galloway and German Angus. They found that a frontal gunshot, placed correctly in the forehead from a distance of 5–15 m, causes brain damage that leads to immediate unconsciousness [26]. It was concluded that the bullet should stay in the animal’s skull to achieve a good stun and a low risk of injury to other animals or there being splinters in the carcass [26]. The research indicated, at least in lighter beef breeds, that this is best achieved with semi-jacketed small-calibre ammunition (.22 Magnum), while larger calibres and full metal jacketed .22 Magnum ammunition tended to pass through the skull. Moreover, blood lactate values were low in animals that had been present in the stun corral earlier the same day that another animal in the group was shot. This suggests that shooting a herd member does not stress other animals kept in the same corral [24]. According to other researchers, however, standard and high-velocity .22 bullets may fail to penetrate the skulls of steers and heifers at the longer distance of 25 m from the shooter [27]. Further research has been deemed necessary to assess the practicalities around the method, test it in more countries and include food-safety aspects.

Full metal jacketed bullets tend to penetrate deep into the tissue or even pass through the body which results in limited tissue damage and haemorrhage, while a semi-jacketed bullet has a tip that deforms upon impact, making the shot more effective by transferring more of the bullet’s kinetic energy to the target. So-called hollow-point bullets expand rapidly on impact. However, they are presently not permitted for stunning slaughter animals in Sweden.

Until recently, EU Regulation (EG) 853/2004 required that healthy domestic ungulates be brought alive to slaughterhouses. Despite this, the gunshot method has been allowed and practised for outdoor cattle in Germany since 2011 [28], provided the transport time from the farm to the slaughterhouse does not exceed 1 h and permission is granted by the responsible regional public authorities. In most of these cases, hollow-point .22 Magnum is probably used (pers. comm. K.J. Schiffer, Swedish University of Agricultural Sciences, 2012). Apart from Germany, the method is permitted and practised in countries such as Switzerland [29], the United States [30], Canada [31], Australia [32] and New Zealand [33]. It is also commonly used for the slaughter of game such as deer.

In April 2021, following public consultation, the European Commission [34] adopted Commission Delegated Regulation (EU) 2021/1374, which amended Annex III to Regulation (EC) 853/2004, to permit the on-farm stunning and slaughter of a limited number of domestic animals, provided that, e.g., a high level of food safety for the meat is maintained and the animals are accompanied by official certification that the hygiene requirements for slaughter have been met. Further research may facilitate the implementation of this regulation.

This study aimed to increase knowledge about the slaughter of cattle by the gunshot method under typical Swedish conditions, i.e., by stunning with a rifle fired at a distance, followed by exsanguination and transport to nearby slaughter premises. We wanted to identify the necessary practical conditions and assess the consequences of the method for animal welfare and food safety.

## 2. Materials and Methods

### 2.1. Farm Conditions and Animals

The study was conducted at a beef farm in central Sweden, selected for its favourable conditions with regard to the size and characteristics of the beef herd, existing infrastructure and owner’s skill and willingness to participate. After considering alternative shooters, the owner himself was appointed shooter. Another person was prepared to replace him if necessary, but was not called in. An experienced and knowledgeable butcher was hired for the exsanguination and slaughter on the farm.

**Figure 1:**
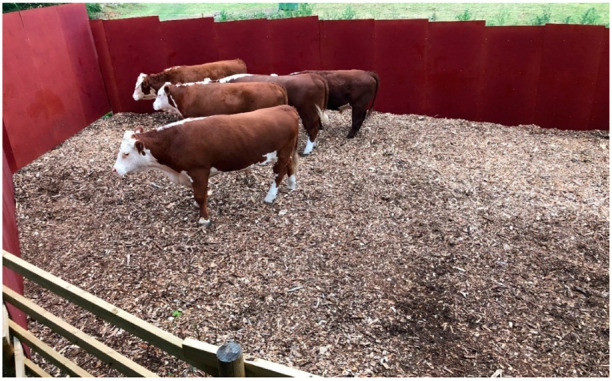
Cattle in the stun corral before gunshot in Sweden, 2020, seen from the shooter’s position 2.9 m above the ground. Photo courtesy of Anne Larsen.

An oval stun corral, 16 × 10 m, was prepared in a more remote location of the farm, next to an existing tall wooden building overlooking the enclosure. The corral had solid wooden walls to a height of 2.40 m including a gate at each end (Figure 1). Immediately behind the corral, a protective earth wall was laid as a backstop. Beef cattle intended for slaughter were grazed in an adjacent open field. The ground surface in the corral sloped slightly away from the building and was covered with wood chips.

Twenty Hereford steers aged 18–54 months were grazed (24 h per day) during the summer until slaughter in September to November 2020. One or two animals were slaughtered every week. On the first two occasions, one animal was shot; although, subsequently, two animals were shot on the same day. The first animal was shot between 7 a.m. and 10 a.m. and the second animal was shot between 9 a.m. and 12 p.m.

### 2.2. Slaughter Process

The entire slaughter process and data collection followed a strict pre-established protocol. However, the height of the shooter position and type of ammunition were modified over the study period to achieve the best possible stun quality.

**Figure 2:**
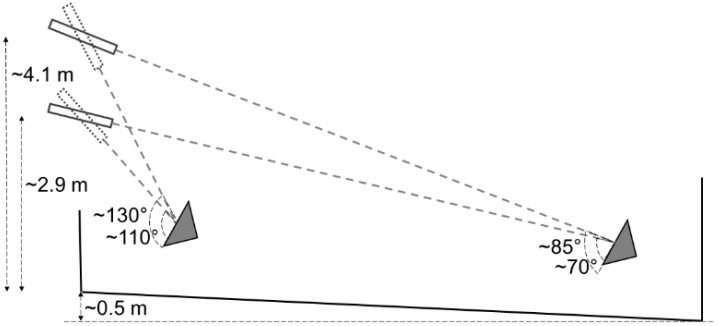
Examples of theoretical shooting distances and impact angles to the forehead at different heights of the rifle and animal during gunshot of cattle in Sweden 2020. The grey triangle represents the animal’s head.

Approximately 1 h before shooting, four to seven animals were gathered in the corral (Figure 1). The shooter was partly concealed in the adjacent building and waited for the right moment for a clean and safe shot at one of the animals. The shooter was instructed to shoot at any one of the animals in the corral and aim at a point on the forehead 2 cm caudally to (above) the cross-section of two straight lines from the horn base to the contralateral outer eye angle. The shot should be in the midline and as perpendicular to the forehead as possible [35]. Depending on the shooter’s position, animal’s location and animal head’s position, the resulting shooting distance and angle to the animal’s forehead varied (Figure 2). For the first eight animals, the rifle was fired from 2.9 m above the ground. Attempting to increase the shooting angle, the height was increased to 4.1 m for the remaining animals. The shooter was standing on a firm platform with elbows resting on an aiming support.

Shooting was done using a hunting rifle (Marlin Firearms, Madison, North Carolina, USA) equipped with a red-dot sight (Acro C-1^TM^ 3.5 MOA, Aimpoint AB, Malmö, Sweden). Two types of small-calibre ammunition (.22 Magnum rimfire) were tested in a systematic order. Twelve animals (#3–#14) were shot with semi-jacketed 3.24 g hollow-point bullets with a muzzle velocity of 511 m/s and muzzle energy of 425 J (.22 Win. Mag. Game-Shok JHP, Federal Premium Ammunition, Anoka, MN, USA) and eight animals (#1–#2 and #15–#20) were shot with 2.59 g jacketed soft-point bullets with a muzzle velocity of 572 m/s and muzzle energy of 423 J (Maxi-Mag Game Point JSP, CCI, Lewiston, Idaho, USA). A Carl Gustaf 2000 hunting rifle (Bofors Carl Gustaf AB, Eskilstuna, Sweden) and large-bore ammunition with a muzzle velocity of 850 m/s and muzzle energy of 3975 J (Norma Tipstrike 30-06 Springfield, 11 g, Norma Precision AB, Åmotfors, Sweden) was used for reshooting. No silencer was used. A cartridge-driven captive bolt gun was kept as a reserve weapon, but never used. After a successful shot and clearance from the shooter, the remaining animals were immediately released from the corral and the shot animal was checked for stun quality.

As soon as possible after stunning, a loader entered the corral and hoisted the shot animal by the hind legs. It was then bled through thoracic sticking, by first opening the skin and then cutting the thoracic blood vessels using two different sterilized knives. The blood was collected in a tub and later discarded. During bleeding, samples were taken for chemical analysis of blood cortisol, glucose and lactate as stress indicators [36,37,38].

**Figure 3:**
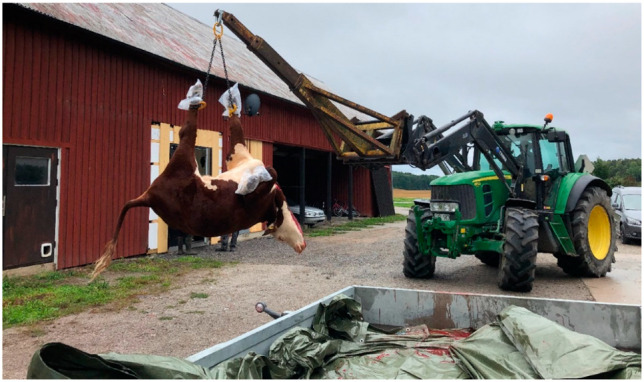
The carcass being hoisted from the trailer and taken to the slaughter premises (in the background) after on-farm gunshot and exsanguination in Sweden 2020. The feet were covered with protected plastic bags. Photo courtesy of Anne Larsen.

**Figure 4:**
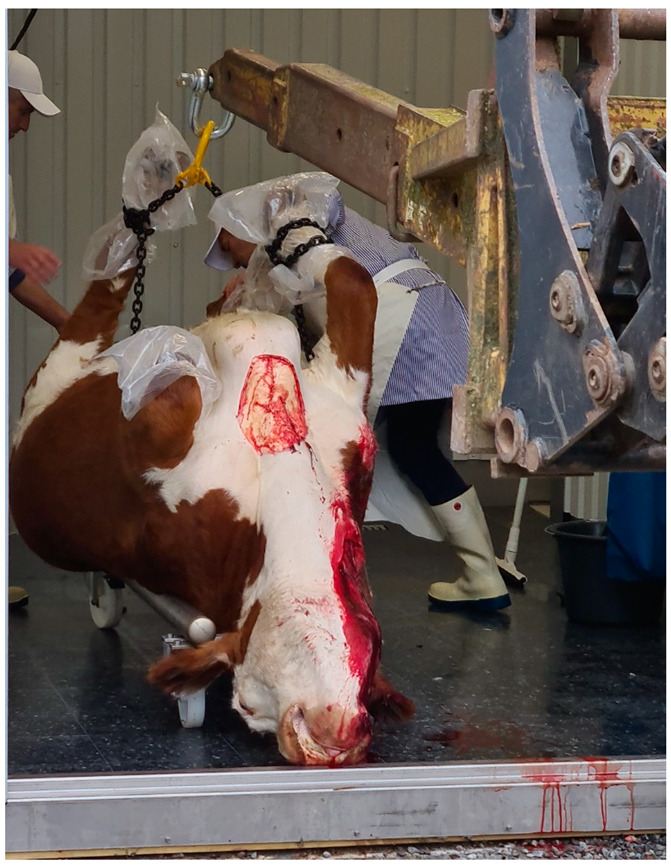
The carcass being placed on a wheeled cradle in the doorway of the on-farm slaughter premises, before removal of hide, head and feet. Photo courtesy of Anne Larsen.

The carcass was placed on an open standard car trailer, wrapped in a clean tarpaulin, the feet covered with protective plastic bags to prevent contamination of the carcass, and transported 2 km to on-farm slaughter premises. Approximately half an hour after stunning, the carcass was lifted off the trailer and onto a wheeled cradle at the entrance to the slaughter premises (Figure 3 and Figure 4), where the first part of the dressing was performed, i.e., removal of hide, feet and head. The dehided head was recovered for measurements and dissection later the same day. Evisceration and further dressing were done with the carcass hanging by the hind legs.

Unclean and clean processes were separated, and only dressed and trimmed carcasses were allowed into the clean part of the slaughter premises. The facility was washed and disinfected before and after each carcass was dressed out. The microbiological quality of the tap water in the facility was checked shortly before the study was started and found acceptable for human consumption.

### 2.3. Data Collection and Analyses

Pre-slaughter conditions and events in the stun corral, as well as the total time the animals were kept in the corral, the time from start of observations to shooting, the stun-to-stick-interval, as well as the time for bleeding, dressing and microbiological sampling, were all recorded by direct observations. Accidentally, the time of entry to the corral was not recorded for animals #1 and #2. The entire stunning and bleeding process was videotaped from the shooter’s location using a portable action camera (GoPro Hero 5 Black, GoPro, Inc., San Mateo, CA, USA) mounted to the wall opening. From the time that the shooter was in position (‘start of recording’) until the remaining animals left the corral, the behaviour of the animals was observed continuously by one person from a concealed location at the corner of the building, approximately 2.5 m above ground level. Behaviours were characterized as walking, listening, exploring or other behaviour (running, turning around, butting, vocalizing, performing flehmen, defecating or urinating). At the start of each recording, the outdoor temperature and relative humidity were measured and the predominant weather conditions were recorded on three binary scales as sunny, windy and rainy. Shooting distance was measured with a portable laser range finder (Strike Inno 600, Optisan Optics Europe GmbH, Berlin, Germany).

Stun quality was assessed in accordance with previous research on captive-bolt stunning of cattle [20,22,39] and a protocol adapted for the gunshot method by Schiffer et al. [26]. Vital behavioural, eye-related and respiratory signs of inadequate stun (Table 1) were noted directly after shooting and the behaviours were later confirmed from the video recordings. The stun was classified as ‘deep’ if: (i) posture, (ii) righting reflex at hoisting, (iii) eye direction and movements, (iv) blinking and (v) corneal reflex were of type a and no other signs were of type c; as ‘poor’ if (i) posture, (ii) righting reflex at hoisting, (iii) eye direction and movements, (iv) blinking, (v) corneal reflex, (vi) breathing or (vii) vocalization was of type c; and as ‘ambiguous’ in all other cases.

Lactate was analysed with portable measuring equipment (Lactate Plus, Nova Biomedical Corp., Waltham, MA, USA), and the median of three separate samples was used. Blood serum was separated, frozen and stored for later chemical analyses of cortisol and glucose at a laboratory. Cortisol was analysed twice with Immulite 2000 (Siemens Healthcare Diagnostics, Erlangen, Germany) and the median value was used. The concentration of glucose was determined with Architect c4000 (Abbott Laboratories, Chicago, IL, USA).

On arrival at the slaughter premises, the trailer with the shot animal and the collected blood was weighed on a weighbridge, to estimate the live weight. In the cases of three animals, a significant amount of blood had been spilled in the stun corral; the weight of the lost blood was therefore estimated subjectively, and the recorded live weight was corrected accordingly. After slaughter, the warm carcass was weighed on a digital slaughter scale (Brecom AS, Årnes, Norway), and the air temperature of the slaughter premises was recorded. Bleeding success was calculated as 100 times the estimated weight of drawn blood divided by the estimated live weight [40].

**Figure 5:**
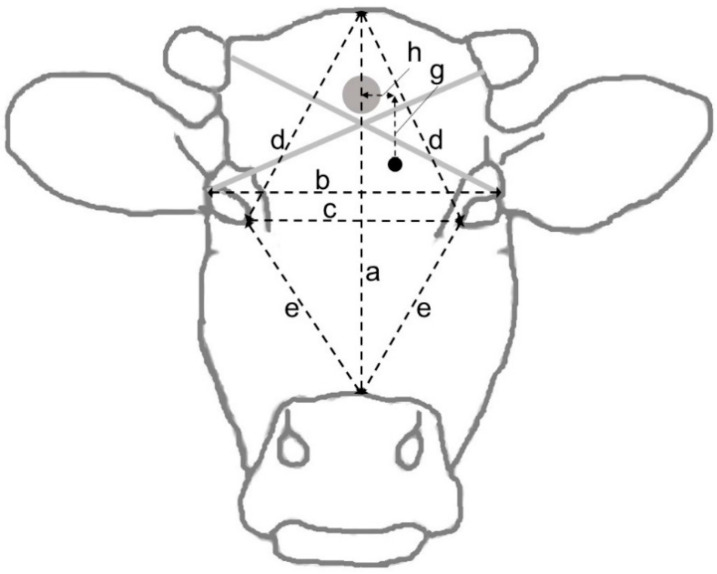
Frontal view of the head showing the different distances measured at dissection following gunshot of cattle in Sweden, 2020. The grey and black dots represent the intended target and an (imaginary) bullet entry point, respectively.

At dissection, the dimensions of the skull and location of the shot (Figure 5), the diameter of the bullet entry hole through the skull, the depth of the bullet canal and the shot angle relative to the forehead rostrally to the hole were measured. The roof of the skull was removed using a saw, and the brain was extracted from the skull and split into hemispheres. The bullet or bullet fragments were recovered, if possible, and weighed, and their positions in the brain were noted. A dorsal photograph was taken of the dehided head. Photographs were also taken of the brain dorsally in situ after removal of the skull roof, ventrally after extraction, and from the medial aspect of both hemispheres after midsagittal section. The measurements of the shot location were later repeated and corrected using the photographs.

In line with previous research [26,35], the extent and degree of lesions and haemorrhages in the brain were assessed from the photographs. Brain tissue damage was characterized as ‘severe’ when there was a complete destruction of the brainstem (composed of the midbrain, the pons and the medulla oblongata), and ‘marginal’ otherwise. Extra-axial brain haemorrhages were assessed on the dorsal and ventral aspects of the cerebrum and the ventral aspect of the brainstem. They were each denoted as ‘moderate’ if covering more than 25% of the visible brain surface, as ‘severe’ if more than 70%, or otherwise as ‘slight’. Intra-ventricular haemorrhages were assessed after midsagittal section and characterized as ‘considerable’ if more than a few small blood clots were found in the ventricles, or otherwise as ‘slight’. The cerebellum, and in some cases a part of the medulla, were not clearly visible in the photographs and therefore not assessed.

The microbiological contamination of carcasses was evaluated after trimming, prior to chilling. The carcasses were sampled by the destructive method, as described by EU Commission Regulation (EC) 2073/2005 and standard ISO 17604:2015 [41]. Four meat samples, approximately 2 mm thick and with a diameter of 2.5 cm, were excised aseptically using a sterile cork drill from the neck, brisket, flank or rump of both carcass halves at approximately 2 cm from the cutting edge. The pooled samples, representing a total area of 20 cm^2^, were placed in a sterile plastic bag and transported to a laboratory for analysis. Total bacterial counts and counts of *Enterobacteriaceae*, *Escherichia coli* and coagulase-positive *Staphylococcus* spp. were calculated as CFU/cm^2^ of sampled area and expressed on the log 10 scale. The presence of two important foodborne pathogens, *Listeria monocytogenes* and verotoxin-producing *E. coli* (VTEC), was analysed but not quantified.

The microbiological samples were put into a blender bag (Grade Packaging, VWR International AB, Spånga, Sweden), mixed with 100 mL of buffered peptone water and stomached in a blender (easyMIX^®^, AES Laboratoire, Combourg, France) for 2 min. Of the resulting primary homogenate, 25 mL was incubated at 41.5 °C for 20 ± 2 h and analysed for VTEC the following day. To analyse *Listeria*, 25 mL of the primary homogenate was mixed with 225 mL of Half Fraser broth (Oxoid, Basingstoke, UK) and incubated at 30 °C for 24 ± 2 h. After incubation, 20 µL of the mixture was plated on Chromogenic *Listeria* Agar ISO plate (Oxoid, Basingstoke, UK) and 20 µL on blood agar (National Veterinary Institute, Uppsala, Sweden), and both were incubated at 37 °C for 48 ± 4 h. Another 100 µL of the same mixture was diluted with 10 mL of Fraser broth (Oxoid, Basingstoke, UK) and incubated at 37 °C for 48 ± 4 h. After incubation, 20 µL was plated on Chromogenic *Listeria* Agar ISO plate and 20 µL on blood agar (National Veterinary Institute, Uppsala, Sweden), and both were incubated at 37 °C for 48 ± 4 h. All incubations were performed under aerobic conditions.

Appropriate serial decimal dilutions of the remaining primary homogenate were made in peptone saline (0.1% peptone in 0.85% NaCl; Dilucups, LabRobot Products AB, Stenungsund, Sweden) and 0.1 mL of each dilution was plated on 3M™ Petrifilm™ (3M Health Care, Eden Prairie, MN, USA) and incubated at 30 °C for 72 ± 6 h for total aerobic bacteria, at 37 °C for 24 h for *Enterobacteriaceae* and at 44 °C for 48 h for *E. coli*. Of each dilution, 0.1 mL was plated on Baird Parker agar (Oxoid, Basingstoke, UK) and incubated at 37 °C for the quantification of coagulase-positive *Staphylococcus* spp. Again, all incubations were performed under aerobic conditions.

In order to detect VTEC, DNA from 200 µL overnight incubated primary homogenate was extracted using the EZ1 DNA Tissue kit (Qiagen, Hilden, Germany) and analysed for verotoxin genes *stx1* and *stx2* and virulence genes *eae* and *saa*. Genes *stx1*, *stx2* and *eae* were detected using the polymerase chain reaction (PCR) with primers and probes according to ISO/TS 13136:2012. The PCR reactions consisted of PerfeCTa qPCR ToughMix with Low ROX (Quantabio, Beverly, MA, USA), 13.3 nM of each *eae* primer, 26.6 nM of each *stx* primer and 100 nM of *eae*, *stx1* and *stx2* probe and 2 µL sample extract, amounting to a total reaction volume of 15 µL. The probes were labeled with HEX, CY5 and FAM fluorophores, respectively. For detection of *saa*, primers and probe were used according to the method of Nielsen & Andersen [42]. The PCR reactions consisted of PerfeCTa qPCR ToughMix with Low ROX (Quantabio), 500 nM of each primer, 100 nM of FAM-labelled probe and 2 µL of sample extract, amounting to a total reaction volume of 15 µL. All PCR analyses were performed using an ABI 7500 Fast Thermocycler (Life Technologies, Carlsbad, CA, USA) and the following thermal profile: 50 °C for 10 min, 95 °C for 3 min and 45 cycles of 95 °C for 3 s and 60 °C for 30 s.

Standard descriptive statistics were produced using Excel 2016 (Microsoft Corp., Redmond, Washington, DC, USA) and Stata IC 15 (StataCorp, College Station, TX, USA). Shot precision, stun quality, levels of stress-related blood constituents, tissue damage, brain haemorrhages and bacterial findings on the carcass were described in detail, looking for possible associations with the recorded animal and environmental factors. The association of stun quality with shooting order on the same day, age, live weight, time from entry to shot, shooting distance, ammunition, shot deviation, skull thickness, brainstem haemorrhage and blood lactate was checked with Spearman rank correlation.

## 3. Results

### 3.1. General Observations

The farmer and two farm employees performed the necessary tasks around the stunning and transporting of the dead animals to the slaughter premises. The estimated animal live weight was 460–900 (median 570) kg. The weather was windy in 6 cases (calm in 14), sunny in 4 cases (cloudy in 16) and it was raining lightly in 2 cases (without precipitation in 18). The outdoor temperature at the start of recording was 2–15 °C (median 12 °C), and the relative humidity 38–100% (median 94%).

### 3.2. Animal Welfare

Table 2 shows the time between the different events around stunning and bleeding. The time from the start of recording to the shot was 1.3–34 (median 5.7) min, and from the shot to sticking was 49–162 (median 100) s. The shooting distance was 6–12 (median 9) m.

The studied animals generally behaved calmly, and no marked fear reactions were observed in any of the animals present, either in connection with the shooting or later. At the shot, they usually displayed a mild startle response, in some cases followed by curiosity about the shot animal. The most commonly observed behaviour in the corral was walking, which was seen on average 3.5 times per animal from the start of recording to their exit from the corral (Table 3).

Two animals were reshot and therefore could not be assessed completely for the stun quality, tissue damage or brain haemorrhage resulting from the first shot. Of the remaining eighteen animals, one displayed an incomplete collapse, seven showed type b kicking while lying and seven showed type b kicking while being hoisted, six showed a type b and one a type c righting reflex at hoisting, ten showed a type b and one a type c reaction to skin cutting, and three showed a type b and one a type c reaction to sticking. Twelve animals had uncertain tail tension and two animals uncertain ear tension. In eight animals, the tongue was not hanging out of the mouth, but in no case was it stiff or moving. The final overall stun quality was classified as ‘deep’, ‘ambiguous’ and ‘poor’ in eleven, six and one of the eighteen animals, respectively.

Mean blood concentrations of cortisol, glucose and lactate were 22 nmoL/L, 3.9 mmoL/L and 2.0 mmoL/L, respectively, varying from <10 to 96 nmoL/L, from 3.5 to 4.6 mmoL/L and from 0.63 to 3.4 mmoL/L, respectively.

Table 4 shows head dimensions and measures of bullet tracks recorded at head dissection. The diameters of the bullet entry holes and the thicknesses of the skull around the holes varied substantially (from 4 to 14 mm and from 3 to 36 mm, respectively). In 5 animals, the skull was thicker than 10 mm. Skull thickness was unrelated to animal age, bodyweight or shot deviation horizontally or vertically. In some cases, the skull roof consisted of several layers of bone tissue, with cavities between them, which made it difficult to obtain reliable estimates of its thickness. The measurements did not provide support for the presence of a bone ridge in the midline. The median angle of the bullet canal to the forehead increased from 75 to 83 degrees after the shooter’s position had been raised. At dissection, it was found that the bullet had changed direction to a flatter angle when passing through the skull roof in some of the skulls. Thus, in animals #17, #18 and #19, the angle was estimated to change from 83 to 55, from 87 to 67 and from 92 to 55 degrees, respectively. In addition, during shooting, it was noted that some shots did not hit the animal straight from the front but slightly from the side; however, it was not possible to quantify the deviation. Bullets or bullet fragments were recovered from the head in 12 cases and weighed between 0.16 and 2.6 (median 1.5) g. There were no obvious associations between stun quality and shot distance, deviation or angle to the forehead.

All animals had marginal brain tissue lesions. Haemorrhages around the brainstem were characterized as moderate in seven animals and severe in 13 animals. Extra-axial haemorrhages observed on the dorsal brain surface were classified as slight in 16 animals, moderate in 4 and severe in none, while haemorrhages on the ventral surface were slight in 5 animals, moderate in 11 and severe in 4. Ventrical haemorrhages were found in 14 animals. There were no obvious associations between brain haemorrhages and shot distance, deviation or angle to the forehead.

### 3.3. Food Safety

Table 5 shows measures related to carcass dressing and food safety. The bleeding lasted for an average of 8 min and the blood drawn accounted for 3.1% of the estimated live weight. All animals were judged to be clean when slaughter commenced, and no faecal contamination of the carcasses was observed. Neither *Enterobacteriacae*, *E. coli*, staphylococci nor VTEC were found, but *Listeria monocytogenes* was found on one carcass.

### 3.4. Results for Individual Animals

Key statistics on animal welfare and food safety measures for individual animals are given in Table 6. When comparing animals with a deep stun with those with an ambiguous or poor stun (including the two reshot animals), 27% vs. 67% were number two in the day, 45% vs. 78% were shot with hollow-point ammunition, 64% vs. 67% had severe brainstem haemorrhages, mean live weight was 611 vs. 588 kg, time from entry to shot was 62 vs. 47 min, shooting distance was 9.1 vs. 7.9 m and deviation from the intended target was 21 vs. 26 mm. None of the rank correlations with stun quality was statistically significant (*p* > 0.05). In animal #8, which was reshot, the shot deviated the most (58 mm) and the skull at the hit location was the thickest (36 mm). When comparing hollow-point with soft-point ammunition (excluding the two reshot animals), 50% vs. 88% had severe brainstem haemorrhages and 50% vs. 75% had a deep stun.

**Figure 6:**
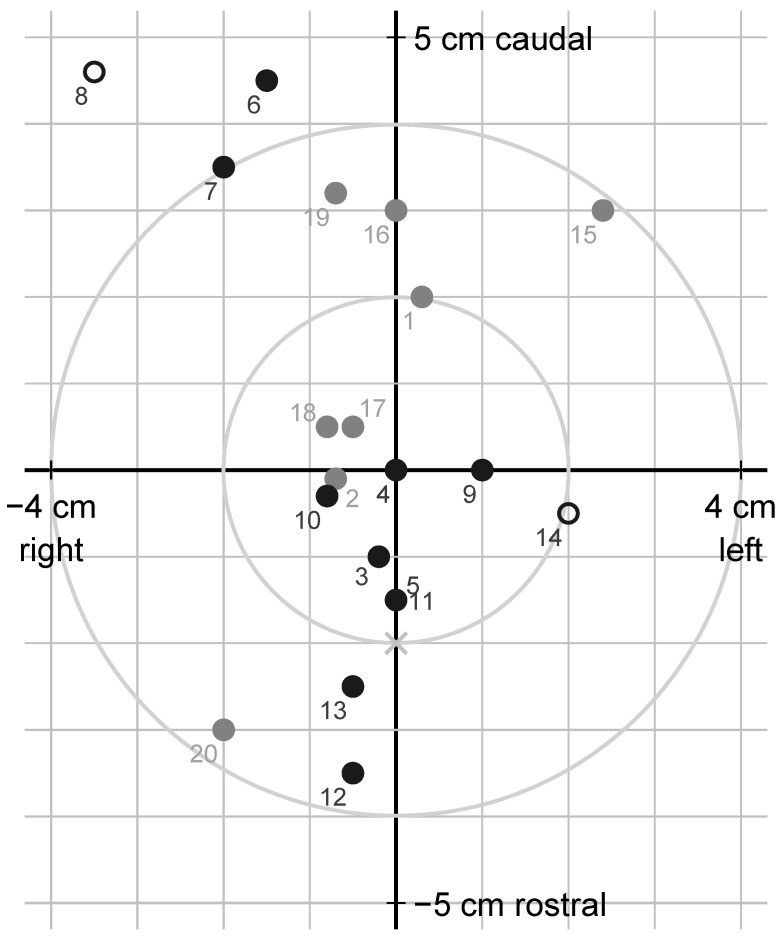
Schematic frontal view of the animal’s forehead at gunshot of cattle in Sweden, 2020, with hit locations relative to the intended target (origin) 2 cm caudally to the cross-section (x) of two straight lines from the horn base to the contralateral outer eye angle; using jacketed soft-point (grey) or semi-jacketed hollow-point (black) ammunition; circles represent reshot animals; marker labels are animal numbers.

Bullet holes relative to the target are shown in Figure 6. The shot was placed an average of 5.7 mm above (caudal to) and 4.0 mm left (on the animal’s right side) of the intended point of impact, from 35 mm below to 46 mm above and from 35 mm left to 24 mm right of it. The shot deviated between 0 and 58 (median 20) mm from the intended target and more than 40 mm in three animals. When asked after the completed data collection, the shooter stated that it was difficult to apply the given instructions, and that he therefore formed his own opinion about a suitable target based on the appearance of the animal’s ears, which also turned out to result in well-placed shots. He also deliberately tried to avoid placing the shot exactly in the midline of the forehead, because he assumed that a bone ridge made the skull thicker centrally, and that this might negatively influence the effect of the shot. According to the shooter, it was difficult to achieve perfect shooting conditions in practice, and, therefore, necessary to weigh different aspects against each other, for example, to accept a slightly flatter shot angle or a shot slightly from the side if it was otherwise possible to fire safely within a reasonable time.

Animal #5 was shot with hollow-point ammunition and collapsed at the shot, but lifted its head while being hoisted and at sticking. Hence the stun was classified as poor. Still, the animal was not reshot. The blood concentrations of cortisol, glucose and lactate during the bleeding of animal #5 were below 10 nmoL/L, 3.6 mmoL/L and 3.4 mmoL/L, respectively, which did not indicate any stress response.

Animal #8 was shot with hollow-point ammunition, and did not collapse on the first shot, which deviated 58 mm from the intended target. The bullet did not completely penetrate the skull roof, but got stuck in it, while a fragment apparently ricocheted and hit a front leg. The animal was reshot after 50 s obliquely from behind on the left side of the head while standing up. The stun-to-stick time was 162 s. The skull thickness around the bullet entry hole from the first shot was 36 mm. Despite the hassle, the blood concentrations of cortisol, glucose and lactate during the bleeding of animal #8 were 12.5 nmoL/L, 3.9 mmoL/L and 1.5 mmoL/L, respectively, which did not indicate any stress response. After the second shot, the haemorrhage around the brainstem was moderate.

Animal #14 collapsed at the first shot with hollow-point ammunition 21 mm from the intended target, but then displayed head movements. The animal was reshot after 32 s in the head obliquely from the front, while it was lying on its chest. Judging from the video, it is doubtful whether reshooting was required. The skull around the bullet entry hole from the first shot was 7 mm thick. The blood concentrations of cortisol, glucose and lactate during the bleeding of animal #14 were below 10 nmoL/L, 3.7 mmoL/L and 1.8 mmoL/L, respectively. Thus, not even in this case did the blood test reveal any stress response. After the second shot, the haemorrhage around the brainstem was severe.

## 4. Discussion

This study shows that the gunshot method can work well, although multiple factors may influence its effectiveness and practical applicability. Skilled staff, proper facility design and adequate ammunition are crucial requirements. The study also shows that good animal welfare and food safety can be achieved if the method is applied correctly. However, the study does not allow direct comparisons between different cattle breeds or other distinct animal categories, animal handling routines, shooting distances, impact angles, transport times to the slaughter facility or dressing procedures. The variation between different circumstances can be substantial and it may not always be possible to reduce it by strict routines.

### 4.1. Animal Welfare

The studied animals were generally calm, with no signs of marked fear or stress, which accords well with Schiffer [24]. Gruber et al. [43] found considerably higher blood glucose and lactate values in cattle exposed to preslaughter stress. Schiffer [24] reported glucose values of 3.2 and 4.4 mmoL/L in two different studies of the gunshot method, and corresponding lactate levels of 2.1 and 3.9 mmoL/L, thus finding values to be significantly lower than at ordinary slaughter at an abattoir. Gruber et al. [43] reported means of glucose and lactate of 12.5 and 12.0 mmoL/L, respectively, at large-scale slaughter; Probst et al. [44] found medians in three trials of 4.9–6.8 and 3.4–3.8 mmoL/L, respectively, in animals treated with gentle touching during 5 weeks before slaughter. In comparison, the median values of glucose and lactate in this study were 3.9 and 2.0 mmoL/L, respectively, with maximums of 4.6 and 3.4 mmoL/L, i.e., in the same order of magnitude as those found by Schiffer [24]. Reports of blood cortisol in cattle at slaughter are less common. Probst et al. [44] found median levels of 106–108 nmoL/L following gentle treatment, and we have previously found values around 70 nmoL/L at on-farm mobile slaughter (unpublished data). In comparison, the median in this study was only 14 nmol/l, and the maximum 96 nmoL/L. The results indicate that the animals had low or very low stress levels when they were shot. The two reshot animals did not show elevated blood levels, but this cannot be taken as a sign of an absence of stress, as the short time to reshooting probably did not allow detection of the physiological reaction. The reason for the relatively large variations in cortisol and lactate values between the studied animals is unknown.

The number of ambiguously or poorly stunned animals may appear high, but the way in which stun quality was assessed differed from practice, and more subtle signs of a poor stun are probably not noticed at all during ordinary large-scale slaughter. In fact, the signs categorized by us as ambiguous may have remained undetected at a commercial slaughterhouse. The shot animal could not be examined closely before remaining animals had been released, 17–74 s later, and brief signs of consciousness may thus have passed unnoticed. However, all behavioural signs were detected from the video recordings. It was not possible to determine the causes of poor stuns with certainty. Animals with ambiguous or poor stun quality were usually the second animal shot on the same day. This might indicate that the animals or the shooter were affected by the shooting of the first animal in a way that increased the risk of a poor stun.

According to Algers and Atkinson [45], tissue damage, including haemorrhages, in the frontal regions of the brain are not necessarily directly related to the depth of the stun, i.e., the stun quality. This is because the arteries enter the brain at its base, which makes the area around the brainstem crucial for obtaining a proper stun. Brain haemorrhages tend to occur on the opposite side of the impact, i.e., as a contrecoup injury [46], and arterial bleedings in the subdural or subarachnoidal region around the brainstem and basal parts of the brain are the most important for a proper stun [44,46,47]. At least moderate haemorrhages around the brainstem are assumed to be required for a good stun [26,45,47,48]. We could not demonstrate a clear link between a deep stun and severe brainstem haemorrhages, compared to moderate haemorrhages, and hence moderate haemorrhages appear to be sufficient from an animal welfare point of view.

The semi-jacketed hollow-point bullet appears to be the most readily available and internationally most commonly used version of .22 Magnum ammunition. For example, the previous German studies [26] used semi-jacketed hollow-point .22 Magnum ammunition, and the soft-point version was not even considered. When testing five types of ammunition, the authors found the hollow-point bullet to be the only one that always stopped at the base of the cranial cavity, instead of passing right through the head. In all thirteen studied animals, it produced brain haemorrhages classified as ‘sufficient’, and in all but one animal it caused a deep stun. It was therefore considered to be favourable from both effectiveness and safety perspectives. However, it should be noted that the sample size was limited. In this study, the stunning success was comparable to the German study. However, we cannot exclude the possibility that another calibre would have resulted in a more effective stun. Hollow-point ammunition produced severe brainstem haemorrhages in five and deep stuns in five out of ten studied animals, while the soft-point resulted in severe haemorrhages in seven and deep stuns in six out of eight animals. In addition, animals with ambiguous or poor stun quality were most often shot with hollow-point ammunition. Although the association was not statistically significant, it may indicate that soft-point ammunition is preferable to hollow point ammunition.

Just like Schiffer et al. [26], we defined the intended target based on the recommendations of Anil and Lambooij [49], Gilliam et al. [50] and Kohlen [51]. Schiffer et al. [26] found that 33% of the shots missed the intended point by more than 4 cm. In comparison, we found 15% of the shots to deviate more than 4 cm. Schiffer et al. [26] recommended that the shooter should be able to hit within 2 cm from the intended target, and the same target size was recommended for effective captive-bolt stunning by Gregory et al. [20], Atkinson et al. [22] and Ilgert [52]. In this study, more than half of the shots did not meet this requirement, and in the study by Schiffer et al. [26], the proportion was even larger. In contrast, Schiffer et al. [35] found all 11 frontal shots on intact cattle heads *postmortem* (animals stunned electrically), i.e., a stationary target, to be within 4 cm from the intended target. This may suggest that it is too demanding in practice to hit a target with a radius of 2 cm in freely moving animals outdoors. Still, in the animal where the shot deviated the most (58 mm), the main part of the bullet got stuck in the skull roof and the animal had to be reshot, confirming that a correct hit is essential. Despite the shooter’s preferences, this study does not justify a re-evaluation of the optimal target when shooting cattle frontally at a distance.

Compared with the animals studied by Schiffer et al. [26,35], the Hereford steers in this study were considerably larger. Head measures indicated that the heads were on average approximately 10–15% larger in this study and the carcasses were approximately 26% heavier. The bullet holes in the skull were also considerably thicker in the present study; Schiffer et al. [26,35] found mean thicknesses of 1.7 and 1.5 mm, respectively, whereas we calculated a mean of 9.9 mm. We also found skull thicknesses to vary substantially, between 3 and 36 mm, and exceeding 14 mm, in five animals, while Schiffer et al. [26,35] found only modest variation between animals. The apparent lack of a clear relationship between skull thickness and shot location suggests that the forehead bone was irregularly shaped, with alternating thick and thin areas, or had cavities, i.e., several layers of bone. It is not known whether this is a breed characteristic, and the subject merits further investigation. Mature bulls, regardless of breed, have thicker skulls than other cattle and may therefore be more difficult to stun properly, but were not included in this study.

A perpendicular angle of impact is usually assumed to be the best way to achieve an adequate stun [53]. Schiffer et al. [26] found a median angle of impact of 85 degrees, and the angle exceeded 100 degrees in 4 out of 30 animals because these animals lowered their heads when eating. The shooter was placed 2 m above ground, and the rifle may have been fired at about 3 m. In this study, we used a corral of approximately the same size; the shooter was placed 2.9 or 4.1 m above ground; and the median angle of the bullet canal was 81 degrees, but in no case more than 92 degrees. Apparently, the shooter’s position, even after being raised, made it difficult to hit the forehead at a right angle, as measured in the medial plane. Regardless, the exact angle did not seem to have a strong effect on the brainstem haemorrhage or stun quality. On the other hand, shots slightly from the side instead of straight from the front might have contributed to ambiguous or poor stun qualities. Interestingly, in some cases, the bullet was found to have changed direction when penetrating the skull roof even though it entered at an angle close to 90 degrees. Schiffer et al. [26] reported a mean penetration depth of 10.2 cm for the semi-jacketed hollow-point .22 Magnum, which accords well with the 10.0 cm found in this study. However, bullet fragments were not always recovered after slaughter, which may indicate that the true lengths of the bullet canals were longer than those measured.

### 4.2. Food Safety

A complete bleed-out is important for meat quality. In this study, the weight of the collected blood was on average 3% of the live weight after 486 s of bleeding, which can be considered a good bleed-out. Anil et al. [40] found the blood to account for 3.1% of the live weight after anesthesia with a bolt gun and 120 s of bleeding.

Outdoor stunning and bleeding may increase the risk of contamination of the carcass, and, as a consequence, compromise the slaughter hygiene. This, however, can be questioned, as in many cases it might be cleaner outdoors than in cramped indoor spaces. Common sources of microbiological contamination of a carcass on a slaughter line include faecal contamination at dehiding or leakage of intestinal content. Hence *E. coli* and *Enterobacteriaceae* on the surface of a carcass are considered useful indicators of bacterial contamination [54]. The total count of aerobic bacteria is used as an overall measure of slaughter hygiene, whereas coagulase-positive *Staphylococcus* spp. indicates poor personal hygiene when handling the carcasses [55]. Monitoring of the total bacterial counts and *Enterobacteriaceae* is required at slaughterhouses in the EU according to Commission Regulation (EC) 2073/2005. The counts of aerobic bacteria, *Enterobacteriaceae* and *E. coli* on the carcasses vary between slaughterhouses, depending on animals’ cleanliness, slaughter procedures (such as decontamination) and other factors, and are generally higher than those found in this study [54,56,57]. According to the EU requirements specified in Commission Regulation (EC) 2073/2005, daily means of less than 3.5 log CFU/cm^2^ of total aerobic bacteria and 1.5 log CFU/cm^2^ of *Enterobacteriaceae* demonstrate a satisfactory hygienic status of cattle carcasses. In Swedish slaughterhouses, coagulase-positive staphylococci were detected on 9% and 16% of cattle carcasses from low- and high-capacity slaughterhouses, respectively [58].

The microbial contamination of the carcasses in our study was low. Our findings suggest good slaughter hygiene and little or no faecal contamination. One carcass was found positive for *Listeria monocytogenes*, which is a widespread ubiquitous bacterium. The prevalence of *Listeria* on cattle carcasses at Swedish slaughterhouses was 1% when using a similar sampling method [56], but can reach as high as 43% when a larger area on the carcasses is sampled [59]. Similar to total bacterial counts, the occurrence of VTEC on carcasses also depends on several factors, including the sampling and method of analysis. An extensive Swedish study reported a prevalence of 2% when using a method similar to this study [56]. A Danish study of a similar scope found a prevalence of 3.4% with real-time PCR following culturing, but a 45% prevalence was found with duplex real-time PCR of enrichment cultures [60]. The prevalence of VTEC on Swedish cattle farms is high [61]. Taken together, the results regarding the bleed-out and carcass contamination indicate that the studied method did not compromise slaughter hygiene and food safety.

### 4.3. Study Design

The study was performed on a selected farm, and the slaughter procedure was adapted during the study, attempting to secure a good stun. Under less optimal on-farm conditions—for example, with an inappropriately designed stunning corral, nervous animals, significant disturbances during shooting or a less experienced and skilled shooter—the resulting animal welfare might have been poorer. Existing on-farm slaughter premises were used for the carcass dressing. Transfer to a more remote slaughterhouse, as well as higher ambient temperatures, may have increased bacterial growth, thus generating higher total carcass counts. Theoretically, a very large number of factors could influence the outcome of the studied method, and the variation between different contexts where the method may be applied is probably substantial. Therefore, although a larger number of study animals may have enabled a more elaborate statistical analysis, the external validity would still be low without studying a variety of farm conditions.

## 5. Conclusions

In conclusion, this study provides a proof-of-concept that the gunshot method is a feasible stunning technique for steers of a medium-size beef breed. The method can maintain a satisfactory level of animal welfare and food safety, provided that the necessary conditions are attained regarding experienced and skilled staff, the proper design of facilities for handling, stunning and dressing, adequate ammunition and weapon, and a swift transport to a slaughter facility for dressing and further processing. We have also shown that it is possible to dress the carcasses in basic on-farm slaughter premises without compromising the slaughter hygiene when adequate slaughter routines are maintained. Because the method is slow and time consuming, it is only applicable to small-scale production.

## Figures and Tables

**Table 1:** Protocol for the assessment of stun quality, following gunshot, of cattle in Sweden, 2020.

Sign	Type a	Type b	Type c
Posture	Immediate collapse	Delayed or gradual collapse	Incomplete or transitory collapse
Kicking after collapse	No kicking	Uncoordinated or slight kicking	Forceful and coordinated kicking
Kicking at hoisting ^a^	No kicking	Uncoordinated or slight kicking	Forceful and coordinated kicking
Righting reflex ^b^ at hoisting ^a^	No movements	Head lifted briefly once during first 20 s	Body curved or head lifted repeatedly or persistently
Movements at skin opening for sticking	No movements	Twitching a little	Forceful kicking, lurching or wriggling
Movements at sticking	No movements or slight twitch	Twitching, kicking, or slight head movement	Forceful kicking, lurching or wriggling
Tail tension	Relaxed	Unclear or slight movements	Clear repeated movements
Ear tension	Completely relaxed	Unclear	Stiff or angled
Tongue tension	Relaxed, hanging out of mouth	Not visible (in mouth)	Stiff, twisted or moving
Eye direction and movements	Wide open, forward, still	Rotated, showing sclera or nystagmus	Directed towards objects
Blinking	No spontaneous blinking	Single blink directly after shooting	Repeated blinking
Corneal reflex ^c^	No corneal reflex	Corneal reflex directly after shooting	Corneal reflex at hoisting ^a^
Breathing	No breathing	Single breath or sigh	Rhythmic and regular breathing
Vocalization	No vocalization	Single sigh	Mooing, panting, moaning or bellowing

^a^ During hoisting or while hanging hoisted before sticking. ^b^ Reflexive movements that correct the orientation of the head or body when it is taken out of its normal upright position. ^c^ Reflexive eye blinking upon touching of the cornea.

**Table 2:** Time intervals between different events during stunning of cattle with the gunshot method in Sweden, 2020.

Time Interval	*n*	Mean	Minimum	Median	Maximum
Entry ^a^ to start of recording, min	18 ^b^	46.2	20.7	43.0	89.3
Entry ^a^ to shot, min	18 ^b^	55.5	22.5	57.0	90.6
Start of recording to shot, min	20	9.76	1.32	5.72	33.9
Shot to sticking, min	20	1.68	0.82	1.66	2.70
Shot to exit ^c^, min	20	0.55	0.28	0.44	1.23
Entry ^a^ to exit ^c^, min	18 ^b^	56.0	23.7	57.4	91.4

^a^ When the animals entered the corral. ^b^ The time of entry to the corral was not recorded for animals #1 and #2. ^c^ When the remaining animals left the corral.

**Table 3:** Frequency of different animal behaviours ^a^ in the stun corral at gunshot of cattle in Sweden 2020; *n* = 20.

Behaviour	Mean	Min.	Median	Max.
Walking ^b^	3.46	0.4	2.25	9.8
Listening	2.50	0	1.70	6.8
Sniffing with head raised	0.398	0	0.171	2
Exploring	2.12	0	1.51	10
Other behaviour ^c^ or inactive	2.41	0.5	2.45	7
Any of the recorded behaviours	10.9	3.43	8.73	27.4

^a^ Number of times the behaviour was seen divided by number of animals in corral from start of recording to exit from corral. ^b^ Taking one or several steps forward or backward, after standing still. ^c^ Running, turning around, butting, vocalising, flehmen, defecating or urinating.

**Table 4:** Measures of head dimensions and bullet tracks recorded at head dissection after gunshot of cattle in Sweden, 2020; *n* = 20.

Measure ^a^	Mean	Minimum	Median	Maximum
Crown to muzzle (a), cm	48.3	43.5	47.5	58.0
Between lateral eye corners (b), cm	24.7	22.0	24.3	28.0
Between medial eye corners (c), cm	21.0	18.0	21.0	24.0
Crown to medial eye corner (d), cm	26.2	23.5	25.8	30.0
Medial eye corner to muzzle (e), cm	25.8	22.0	25.8	30.0
Vertical deviation ^b^ (g), mm	−24	−80	−33	51
Horizontal deviation ^c^ (h), mm	−4	−35	−5	24
Bullet hole diameter, mm	7	4	6	14
Bone thickness in bullet hole, mm	10	3	4	36
Angle of shot to head ^d^, degrees	81	70	81	92
Penetration depth, cm	10.0	2.8	10.3	14.5

^a^ Letters within parentheses refer to Figure 5. ^b^ Vertical distance between the intended target to the actual hit location, caudally (towards the crown). ^c^ Horizontal distance between the intended target to the actual bullet entry point, towards the animal’s left side. ^d^ Measured at bullet entrance, rostrally to the entry point (towards the muzzle).

**Table 5:** Measures relating to carcass dressing and food safety at gunshot of cattle in Sweden, 2020; *n* = 20 ^a^.

Measure	Mean	Minimum	Median	Maximum
Time for bleeding, min	8.1	5.0	7.7	11.4
Time from shot to dressing, min	26.8	20.7	26.6	35.4
Time from shot to bacterial sampling, min	104	82.7	100	144
Time from dressing to bacterial sampling, min	77.6	56.8	73.8	110
Warm carcass weight, kg	315	228	290	505
Dressing percentage, %	52	48	52	56
Bleeding success ^b^, %	3.1	2.2	3.1	4.0
Temperature in slaughter premises, °C	14	7	14	19
Total bacterial count ^c^, logCFU/cm^2^	1.4	0	1.6	2.6

^a^ For temperature in slaughter premises, *n* = 18. ^b^ Estimated drawn blood weight divided by estimated live weight. ^c^ Total count of aerobic bacteria on carcass surface; no detected bacteria scored as 1 CFU/cm2 (CFU = colony-forming units).

**Table 6:** Key statistics for individual animals at gunshot of cattle in Sweden, 2020.

Animal no.	Shooting Order ^a^	Age, Months	Live Weight, kg	Time to Shot ^b^, min	Shooting Distance, m	Ammunition ^c^	Reshooting	Shot Deviation ^d^, mm	Skull Thickness ^e^, mm	Brain Haemorrhage ^f^	Stun Quality ^g^	Stun-to-Stick Time, s	Blood Lactate, mmol/L	Total Bacterial Count ^h^, logCFU/cm^2^
1	1	19	480	-	8	Soft	No	20	10	Moderate	Deep	121	2.4	2.56
2	1	42	646	-	8	Soft	No	7	3	Severe	Ambiguous	123	3.1	1.60
3	1	54	900	76.8	10	Hollow	No	10	3	Moderate	Deep	115	1.8	2.00
4	2	42	770	90.6	11	Hollow	No	0	4	Moderate	Deep	117	1.7	1.40
5	1	19	460	61.1	9	Hollow	No	15	3	Moderate	Poor	85	3.4	2.18
6	2	19	470	55.3	9	Hollow	No	47	3	Severe	Ambiguous	96	2.1	1.60
7	1	19	560	66.8	9	Hollow	No	40	3	Severe	Deep	103	0.6	1.74
8	2	30	655	22.5	7	Hollow	Yes	58	36	Moderate ^i^	Poor ^j^	162	2.5	2.31
9	1	32	580	32.6	7	Hollow	No	10	3	Severe	Ambiguous	121	0.9	1.48
10	2	30	630	30.4	8	Hollow	No	9	3	Severe	Ambiguous	86	1.0	1.88
11	1	19	540	60.7	6	Hollow	No	15	8	Severe	Deep	88	1.9	1.30
12	2	32	680	49.7	9	Hollow	No	35	4	Moderate	Ambiguous	101	2.7	1.88
13	1	20	530	55.1	7	Hollow	No	25	3	Moderate	Deep	89	2.1	0.70
14	2	31	643	65.4	7	Hollow	Yes	21	7	Severe ^i^	Ambiguous ^j^	120	1.7	1.60
15	1	20	480	44.1	10	Soft	No	38	17	Severe	Deep	89	2.5	0
16	2	18	486	61.4	9	Soft	No	30	10	Severe	Deep	98	2.5	0
17	1	21	490	77.9	9	Soft	No	7	27	Severe	Deep	57	1.3	1.40
18	2	32	690	47.2	12	Soft	No	9	32	Severe	Deep	102	2.3	1.54
19	1	45	790	43.2	9	Soft	No	33	4	Severe	Deep	89	1.5	0
20	2	21	530	58.8	7	Soft	No	36	15	Severe	Ambiguous	49	1.8	0.70

^a^ Shooting order on the same day. ^b^ Time from entry into stun corral to shot. ^c^ Soft = jacketed soft-point; hollow = semi-jacketed hollow-point. ^d^ Shot deviation from the intended target, which was located 2 cm caudally to the cross-section of two straight lines from the horn base to the contralateral outer eye angle; in reshot animals, measurements of the first shot. ^e^ Measured in the bullet entry hole; in reshot animals, measurements of the first shot. ^f^ Haemorrhage around the brainstem, classified as slight, moderate or severe. ^g^ Classified as ‘deep’, ‘ambiguous’ or ‘poor’ based on vital behavioural, eye-related and respiratory signs. ^h^ Total count of aerobic bacteria on carcass surface; no detected bacteria scored as 1 CFU/cm^2^ (CFU = colony-forming units). ^i^ Haemorrhage resulting from both shots. ^j^ Stun quality resulting from first shot.

## Data Availability

Data are contained within the article.
